# Regional inequalities in self-rated health and disability in younger and older generations in Turkey: the contribution of wealth and education

**DOI:** 10.1186/s12889-015-2273-5

**Published:** 2015-09-29

**Authors:** Isil Ergin, Anton E. Kunst

**Affiliations:** Department of Public Health, Ege University School of Medicine, Bornova Izmir, Turkey; Department of Public Health, Academic Medical Centre (AMC), University of Amsterdam, Amsterdam, The Netherlands

**Keywords:** Self-rated health, Disability, World Health Survey, Health inequalities, Geographic inequalities, Turkey

## Abstract

**Background:**

In Turkey, large regional inequalities were found in maternal and child health. Yet, evidence on regional inequalities in adult health in Turkey remains fragmentary. This study aims to assess regional and rural/urban inequalities in the prevalence of poor self-rated health and in disability among adult populations in Turkey, and to measure the contribution of education and wealth of individual residents. The central hypothesis was that geographical inequalities in adult health exist even when the effect of education and wealth were taken into account.

**Methods:**

We analyzed data of the 2002 World Health Survey for Turkey on 10791 adults aged 20 years and over. We measured respondents’ rating of their own general health and the prevalence of five types of physical disability. Logistic regression was used to estimate how much these two health outcomes varied according to urban/rural place of residence, region, education level and household wealth. We stratified the analyses by gender and age (‹50 and ≥50 years).

**Results:**

Both health outcomes were strongly associated with educational level (especially for older age group) and with household wealth (especially for younger age group). Both health outcomes also varied according to region and rural/urban place of residence. Higher prevalence rates were observed in the East region (compared to West) with odd ratios varying between 1.40–2.76. After controlling for education and wealth, urban/rural differences in health disappeared, while regional differences were observed only among older women. The prevalence of poor self-rated health was higher for older women in the Middle (OR = 1.69), Black Sea (OR = 1.53) and East (OR = 2.06) regions.

**Conclusion:**

In Turkey, substantial geographical inequalities in self-reported adult health do exist, but can mostly be explained by differences in socioeconomic characteristics of residents. The regional disadvantage of older women in the East, Middle and Black Sea may have resulted from life-long exposure to gender discrimination under a patriarchal ideology. Yet, not geographic inequalities, but the more fundamental socioeconomic inequalities, are of key public health concern, also in Turkey.

## Background

Many studies documented that the health and survival of adults can strongly vary within countries according to region of residence. Regional inequalities in health have been documented in much detail in the United States [1], in western European countries such as United Kingdom [2], France [3], Spain [4] and Italy [5], and in eastern European and former Soviet Union countries [6, 7]. Research that aims to understand such regional inequalities typically distinguish between compositional effects (i.e. effects related to the demographic and socioeconomic composition of regional populations) and contextual effects (i.e. characteristics of the physical, economic and social-cultural environment of each region) [8, 9]. Whereas compositional effects often appear to explain the largest part of regional health inequalities, observed contextual effects may be relevant both practically and theoretically [10].

According to World Bank classification, Turkey is an upper-middle income country with its GDP per capita 10 970 dollars [11]. However, Turkey ranks last in life expectancy at birth for women among 34 OECD countries [12]. Literacy rates are 91 % for women, and 98.3 % for men [13]. In 2013, Turkey was the third most unequal country among OECD countries regarding its Gini coefficient for income distribution [14]. Turkey has large regional inequalities. Regions differ strongly in terms of demographic factors, employment rates, educational levels, infrastructure, level of welfare, and economic structure [15]. There is a huge East–West divide in the development of agriculture and industry, in working and earning conditions, in the potential for public or private investment and in the direction migration flows [16]. Income inequality is most profound in the Eastern parts despite the Priority Provinces in Development programs [17, 18]. The percentage of illiterate women peaks at 34.08 % in the South-east regions while it is 6.30 % in Marmara (Istanbul zone). Social security does not cover 64 % of the population of Eastern regions compared to 35 % in the West [19]. The availability, capacity and quality of public services also show large East–West regional differences [20]. All these differences bring along a highly unequal distribution of opportunities and living circumstances for the residents.

Rural areas differ from urban areas in a similar way as in the East–West divide. Rural municipalities lack financial capacity as their population decreases, resulting in problems in supplying services such as adequate water or wastage disposal. In 2000, the percentage of households which used unimproved/surface water for drinking was 15 % in rural areas (compared to 5 % in cities) while improved sanitation facilities were lacking in 29 % of rural households (compared to 4 % in cities) [21].

Such pronounced geographic differences in living conditions are likely to be reflected in differences in the state of population health. Some Turkish studies have described regional differences in non-communicable diseases and adult health. The Burden of Disease Study in 2005 has suggested that the West, Middle, North and South regions had patterns similar to European countries while the East showed disease and mortality patterns similar to developing countries [22]. As regards to smoking in Turkey, Hassoy et. al revealed that among men, no regional differences in smoking existed in the older men yet smoking prevalence rates among younger men were higher in the West and Black Sea regions. A similar East–West pattern was observed among older women [23]. Sozmen et al. found that inequalities in self-assessed adult health were mainly related to educational level, household wealth and geographical area, thus suggesting an important geographical component [24]. On the other hand, the 17-year cohort of TEKHARF study found no major differences between geographic regions or by rural/urban residence in overall mortality [25] or in cardiovascular mortality [26, 27]. Yet, age-adjusted coronary heart disease incidence rates showed large regional differences, with higher rates among men in the Black Sea (1.89 times), among men in the Marmara Region (1.76 times) and among South-Eastern women (2.07 times) in comparison with the Middle Anatolia Region [27]. The national study of diabetes epidemiology (TURDEP) showed that in 2010 diabetes prevalence was lowest in the North region (14.5 %) and highest in the East region (18.2 %) [28]. A study on socioeconomic inequalities in overweight in Turkey observed small regional variations; the East was found to have an inverse pattern of inequalities corresponding to its low level of socio-economic development [29].

Large regional differences are generally observed for infectious diseases and for maternal and child health. For example, morbidity and mortality in communicable diseases related to inadequate water and food supply (e.g. typhoid fever, giardiasis, brucellosis, anthracis, hidatidosis and parasite diseases) peak in the South-East region [30]. This increased burden however, does not correspond to an increased service delivery or quality in these regions. Vaccination rates are lowest in these areas. Means of prenatal care and services for delivery also vary substantially between regions [31, 32]. Such an unequal delivery of health services may exacerbate geographical inequalities in health rather than compensate for them.

In Turkey, the regional comparisons that focused on adult health indicators have not aimed to explain the inequality patterns that were observed. Thus, socioeconomic determinants are rarely evaluated in scientific studies and monitoring reports, demonstrating the absence of an “equity lens” in public health research and monitoring in Turkey. For example, the recent Health Survey 2012 report of the Turkish Statistical Institute has examined health outcomes according to sex, age and urban/rural place of residence, but did not address regional or socioeconomic factors [33]. This incomplete approach is common in governmental documents as well as scientific works. Yet, assessing the role of socioeconomic factors is essential for any evaluation that aims to support policies aimed at addressing health inequalities in Turkey.

This study aims to assess regional and rural/urban inequalities in general self-rated health and in disability prevalence among adult men and women in Turkey and to measure the contribution of education and wealth. Our key hypothesis was that regional and urban/rural health inequalities do exist even when taking into account differences in the educational level of residents and the wealth of households. This hypothesis will be assessed for men and women in two different age groups.

## Methods

### Data

World Health Survey (WHS) 2002 country data for Turkey has been used in this study. Household face-to-face surveys were used for the data collection in Turkey. According to the official report on the Turkish WHS, the study yielded a response rate of 97.5 % [34]. Permission to use the official data of WHS and to perform the study was given by the WHO. For this study, household questionnaire and individual questionnaire data were used [35]. Of the 11 479 respondents in the individual questionnaire database, we excluded 263 respondents who did not match with the household database. Respondents 20 years and older were selected for analysis (*N* = 10807, excluding 409 individuals younger than 20 years). Individuals for missing data on wealth, disability and self-rated-health (SRH) were excluded from analysis (*N* = 16). The data of the remaining 10,791 respondents were used for analyses. According to evaluations presented in the WHS Turkey report, the age-sex structure is about representative of the general population distribution, but elderly are overrepresented [34].

### Variables

The following independent variables were included in the analysis: age, sex, urban/rural residence, region of residence, household wealth and individual education. Age was measured in years. We constructed two age groups those below 50 who represented “the younger” and those above 50 who were considered “the older” respondents. We aimed to have sufficient number of participants within each age group. A more even distribution was achieved around the age of 50 years as compared to higher ages. Although a cut-off age of 60 or 65 years may be more common in high income countries, a lower age may be more appropriate in countries such as Turkey where life expectancies are lower, and the population structure is still young [36–38].

**Fig. 1:**
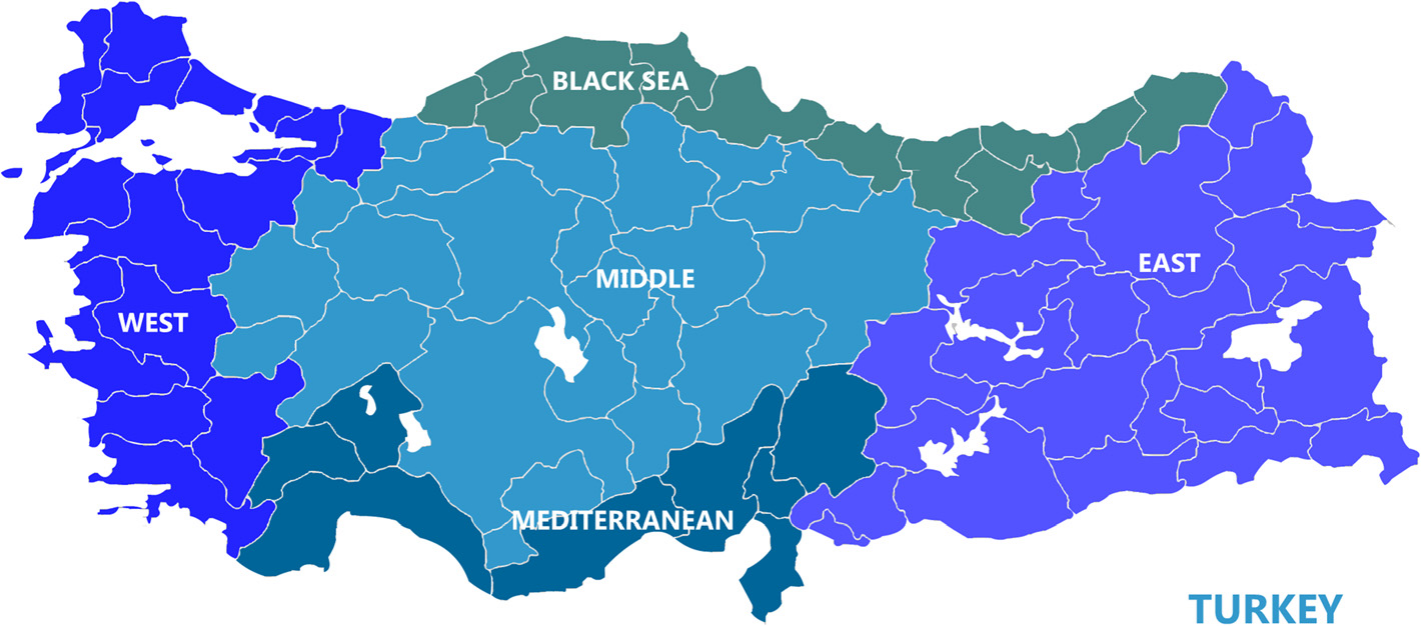
The five regions in Turkey

Residence was coded as “urban” versus “rural” as it was in the original database. In the WHS, five Turkish regions were distinguished: West, Mediterranean, Middle, Black Sea and East (Fig. 1). Using the socioeconomic development scores for cities developed by Turkish State Planning Organization [39], the regions were found to strongly differ. On a scale in which “1” represented the most developed cities and “5” the least developed cities or areas, West is at 1.73, Mediterranean at 2.88, Middle at 3.09, Black at 3.50, and East at 4.65.

Education was asked for as the number of years of education in WHS questionnaire. In this study, the number of years of education was grouped as 0–4, 5–7, 8–10 and 11+ years. This classification corresponds to the Turkish compulsory scheme of education, in which primary education lasts five years, secondary education takes three additional years, and completion of higher levels takes at least another three more years.

For household wealth, questions on household assets have been used. Eleven wealth items have been asked for in the WHS survey: stereo system, washing machine for clothes, washing machine for dishes, vacuum cleaner, refrigerator, fixed line telephone, mobile/cellular telephone, computer, access to the internet, subscriptions to magazines and/or newspaper, and a security system in home. In this study, the responses to these eleven items were scored as 0 (Does not have the item) or 1 (Has the item). The total wealth score for each respondent was calculated as a sum of these item scores. In the case of the missing items, the sum score was multiplied by the ratio of 11/number of items answered. The wealth scores were grouped as 8–11 (highest), 6–7 (second highest), 5 (middle), 4 (second lowest) and 0–3 (lowest). Several alternative grouping have been evaluated, with similar results. In this paper, we present results for this classification as it resulted in a reasonable number of respondents within each wealth group in each region.

The two dependent variables of this study were self-rated health (SRH) and disability. The questions were decided upon by the WHS team at WHO in Geneva [40]. SRH was measured as the people’s perception of their general health which they could rate according to five categories ranging from “very good” to “very bad”. We dichotomized this variable by grouping very bad/bad versus moderate/good/very good rating. For health state descriptions, in addition to including a question on overall health (self-rated health), WHS team added detailed questions on specific physical disabilities related to mobility, self-care, pain and discomfort, cognition, interpersonal activities, vision, sleep, energy and affect. For example, for vision impairment, the question in WHS questionnaire was “In the last 30 days, how much difficulty did you have in seeing and recognizing a person you know across the road (i.e. from a distance of about 20 m)?” Use of vision aids was not asked for. Similar to other items, this question aims to measure actual impairment that cannot be dealt with by just wearing glasses.

The components and the cut-off of the disability score used in our paper have not been operationalized in other work, yet similar efforts had been made previously [41]. For our disability variable, we used ten questions covering five areas (mobility, self-care, cognition, interpersonal activities, and vision) to calculate the disability score. The score on each question ranged from 1 to 5 (1:none, 5: extreme). These scores were summed to obtain the summary disability score, for which the maximum was 50. Scores higher than 20 were marked as ‘high disability’ score, as found this to result in a reasonable number of respondents in both the ‘high disabled’ and ‘other’ group.

### Statistical analysis

Logistic regression was used to estimate differences in these two health outcomes according to urban/rural residence, region of residence, educational level and household wealth. We made separate analyses by gender and by age (‹50 and ≥50 years). Control was made for 5-year age group in the first model. In the second model, all other geographic and socioeconomic variables were also included as control variables. Regression coefficients and their standard errors were used to calculate odds ratios and their 95 % confidence intervals. The most advantaged socioeconomic groups and regions (i.e. urban and the West) were taken as the reference categories.

## Results

In the study group, the mean age was 42.88 years. 57.2 % were women, 45.1 % lived in the West or Mediterranean Region, 69.4 % had primary school education or below, 59.1 % belonged to a wealth group above middle (Table 1). Only 9.8 % defined their health as bad/very bad and 19.1 % got a disability score higher than 20.

**Table 1:** Odds ratios for disability according to residence, region, education and wealth groups (men), WHS, Turkey 2002

	Men (<50 years)					Men (≥50 years)				
	N	Age control^a^		Full control^b^		N	Age control^a^		Full control^b^	
		OR	95 % CI		95 % CI		OR	95 % CI		95 % CI
Residence										
Urban	1494	1.00	-	1.00	-	801	1.00	-	1.00	-
Rural	1532	1.11	0.81–1.54	1.08	0.78–1.50	796	1.27*	1.01–1.60	1.20	0.94–1.53
Region										
West	923	1.00	-	1.00	-	504	1.00	-	1.00	-
Med	375	0.62	0.31–1.25	0.56	0.28–1.14	237	0.89	0.61–1.32	0.77	0.51–1.15
Middle	449	1.27	0.75–2.14	1.09	0.64–1.85	244	1.54*	1.08–2.20	1.23	0.84–1.79
Black Sea	367	0.97	0.53–1.79	0.98	0.53–1.82	201	1.06	0.71–1.59	0.92	0.61–1.40
East	912	1.96**	1.31–2.93	1.40	0.91–2.15	411	1.63*	1.20–2.21	1.17	0.85–1.61
Education										
8+	1479	1.00	-	1.00	-	381	1.00	-	1.00	
5–7 years	1377	2.90**	1.95–4.32	2.34**	1.52–3.58	823	2.67**	1.84–3.88	2.12**	1.43–3.14
0–4 years	170	7.01**	4.08–12.03	4.38**	2.39–8.03	393	5.40**	3.63–8.03	3.52**	2.29–5.40
Wealth										
Highest wealth	749	1.00	-	1.00	-	276	1.00	-	1.00	-
Second highest	1162	1.24	0.73–2.11	0.96	0.55–1.65	578	1.58*	1.03–2.42	1.27	0.82–1.99
Middle	408	2.67**	1.51–4.72	1.80	0.99–3.27	321	2.21**	1.41–3.46	1.61*	1.00–2.58
Second lowest	283	3.45**	1.88–6.35	1.93*	1.01–3.71	172	3.57**	2.19–5.82	2.24*	1.33–3.76
Lowest	424	4.20**	2.47–7.15	1.88*	1.03–3.45	249	5.10**	3.24–8.01	2.81**	1.72–4.59

^a^Age control: the regression includes age as control variable

^b^Full control: the model also includes residence, region, education and wealth as control variables

*p\0.05, **p\0.001

In Table 1 with control for age only, among men, the rural older men had 1.27 times increased odds of disability compared to urban dwellers. Regional differences were to the disadvantage of Eastern men (1.96 times higher odds for young ones and 1.63 times for older) and of older men in the Middle region (1.54 times). These differences were all virtually eliminated after full control for education and wealth. The odds ratios for education and wealth groups showed regular gradients. Controlling for the other variables diminished the effect of education and wealth to only a modest extent. After full control, the lowest education group of younger men had a 4.38 times increased odds and older men had a 3.52 times increased odds for disability as compared to the best-off. For wealth, in the second lowest and lowest wealth groups, the odds of disability were 1.88 and 1.93 times increased among younger men, while it was 2.81 and 2.24 times increased among older men. In these older men, middle wealth groups were also significantly more (1.60 times) disabled after full control.

In Table 2, among women, no urban/rural difference in the prevalence of disability existed after control for age or after full control. After controlling for age only, the prevalence of disability was higher in younger Eastern women (1.40 times) and in older women in the Middle (1.58 times), Black Sea (1.51 times) and East (1.70 times) regions. However, no regional differences remained after full control. The increased odds for lowest education groups was highest for older women (OR = 8.47), but was high as well for younger ones (OR = 5.14). The differences that were observed after control for age only, persisted after full-control. Large differences were also observed for wealth, with 3.69 times higher odds for lowest wealth group in younger women and a 3.26 times higher odds for older. The wealth-related gradient diminished but still existed after full control both for the younger and the older age groups. However, in older women, an increased risk for disability (1.66 times) remained only for the lowest wealth group.

**Table 2:** Odds ratios for disability according to residence, region, education and wealth groups,(women), WHS, Turkey 2002

	Women (<50 years)					Women (≥50 years)				
	N	Age control^a^		Full control^b^		N	Age control^a^		Full control^b^	
		OR	95 % CI		95 % CI		OR	95 % CI		OR
Residence										
Urban	2148	1.00	-	1.00	-	912	1.00	-	1.00	-
Rural	2241	1.06	0.89–1.26	1.04	0.87–1.24	867	1.14	0.94–1.38	1.20	0.98–1.47
Region										
West	1432	1.00	-	1.00	-	598	1.00	-	1.00	-
Med	533	0.61*	0.43–0.85	0.61*	0.43–0.85	270	.97	0.72–1.32	0.86	0.63–1.18
Middle	725	1.12	0.87–1.44	0.99	0.76–1.29	251	1.58*	1.16–2.15	1.38	0.99–1.91
Black Sea	503	0.94	0.69–1.27	0.88	0.65–1.21	226	1.51*	1.10–2.08	1.15	0.83–1.61
East	1196	1.40*	1.13–1.74	0.97	0.77–1.23	434	1.70**	1.31–2.20	1.23	0.93–1.62
Education										
8+	1268	1.00	-	1.00	-	175	1.00	-	1.00	-
5–7 years	2272	2.37**	1.83–3.08	2.04*	1.55–2.67	540	4.34**	2.71–6.95	4.01**	2.49–6.48
0–4 years	849	5.14**	3.89–6.80	3.64*	2.66–4.99	1064	8.47**	5.40–13.30	6.93**	4.31–11.15
Wealth										
Highest wealth	1080	1.00	-	1.00	-	223	1.00	-	1.00	-
Second highest	1724	1.77**	1.37–2.29	1.37*	1.05–1.79	581	1.56*	1.12–2.19	1.16	0.81–1.67
Middle	633	2.28**	1.68–3.10	1.51*	1.09–2.08	418	1.82**	1.28–2.59	1.13	0.77–1.66
Second lowest	392	3.15**	2.26–4.38	1.90**	1.33–2.70	215	2.39**	1.60–3.58	1.31	0.85–2.04
Lowest	557	3.69**	2.74–4.97	1.95**	1.39–2.73	342	3.26**	2.24–4.72	1.66*	1.10–2.50

^a^Age control: the regression includes age as control variable

^b^Full control: the model also includes residence, region, education and wealth as control variables

*p\0.05, **p\0.001

Tables 3 and 4 present odds ratios for SRH for men and women. Among men (Table 3), urban/rural difference was not found. Compared to the West region, young men of the East region (2.06 times) and older men of the Middle region (1.67 times) had increased odds for bad SRH. No regional difference remained after adjustment for all variables. For education, the odds for bad health was increased for the middle and lowest education groups. After full control, the gradient remained although with a diminished gap in both age groups. For wealth, the strong gradient observed for younger men after control for age, persisted after full control, although to a lesser extent. Older men showed increased odds at the second lowest (OR = 2.74) and lowest (OR = 3.90) wealth groups but this effect remained statistically significant only in the lowest group (OR = 1.99) after full adjustment.

**Table 3:** Odds ratios for self-rated-health according to residence, region, education and wealth groups (men), WHS, Turkey 2002

	Men (<50 years)					Men (≥50 years)				
	N	Age contro^a^		Full control^b^		N	Age control^a^		Full control^b^	
		OR	95 % CI		OR		OR	95 % CI		OR
Residence										
Urban	1532	1.00	-	1.00	-	794	1.00	-	1.00	-
Rural	1494	1.12	0.78–1.59	1.08	0.76–1.56	800	1.21	0.87–1.67	1.11	0.79–1.55
Region										
West	923	1.00	-	1.00	1.00	504	1.00	-	1.00	1.00
Med	375	0.95	0.48–1.87	0.84	0.42–1.67	237	1.20	0.70–2.06	1.04	0.60–1.80
Middle	449	1.12	0.61–2.05	0.83	0.45–1.54	244	1.67*	1.02–2.74	1.27	0.76–2.13
Black Sea	367	0.81	0.39–1.67	0.75	0.36–1.56	200	0.94	0.51–1.72	0.81	0.43–1.50
East	912	2.06*	1.32–3.22	1.21	0.75–1.94	409	1.51	0.98–2.34	1.04	0.66–1.64
Education										
8+	1479	1.00	-	1.00	-	380	1.00	-	1.00	
5–7 years	1377	2.45**	1.60–3.75	1.43	0.90–2.25	822	3.82**	1.95–7.48	3.36*	1.67–6.77
0–4 years	170	5.99**	3.35–10.69	2.29*	1.20–4.40	392	7.24**	3.64–14.40	5.04**	2.41–10.52
Wealth										
Highest wealth	749	1.00	-	1.00	-	276	1.00	-	1.00	-
Second highest	1162	2.87*	1.32–6.24	2.53*	1.15–5.57	578	1.01	0.55–1.86	0.74	0.39–1.39
Middle	408	4.78**	2.07–11.02	3.94*	1.68–9.28	321	1.33	0.70–2.53	0.87	0.45–1.71
Second lowest	283	11.18**	4.97–25.16	8.50**	3.63–19.92	171	2.74*	1.42–5.28	1.56	0.78–3.11
Lowest	424	11.54**	5.35–24.86	7.46**	3.24–17.19	248	3.90**	2.14–7.10	1.99*	1.04–3.82

^a^Age control: the regression includes age as control variable

^b^Full control: the model also includes residence, region, education and wealth as control variables

*p\0.05, **p\0.001

**Table 4:** Odds ratios for self-rated-health according to residence, region, education and wealth groups (women), WHS, Turkey 2002

	Women (<50 years)					Women (≥50 years)				
	N	Age control^a^		Full control^b^		N	Age control^a^		Full control^b^	
		OR	95 % CI		OR		OR	95 % CI		OR
Residence										
Urban	2148	1.00	-	1.00	-	868	1.00	-	1.00	-
Rural	2240	1.28*	1.04–1.57	1.26*	1.02–1.56	912	1.03	0.82–1.30	1.05	0.83–1.34
Region										
West	1432	1.00		1.00		598	1.00		1.00	
Med	533	0.52*	0.33–0.83	0.49*	0.31–0.79	270	1.09	0.72–1.65	0.91	0.60–1.40
Middle	724	1.47*	1.09–1.98	1.24	0.91–1.69	252	1.98**	1.36–2.89	1.69*	1.15–2.50
Black Sea	503	0.80	0.53–1.20	0.71	0.47–1.07	226	1.95*	1.32–2.86	1.53*	1.03–2.28
East	1196	1.70*	1.31–2.20	1.05	0.79–1.39	434	2.76**	2.02–3.78	2.06**	1.48–2.85
Education										
8+	1267	1.00	-	1.00	-	175	1.00	-	1.00	-
5–7 years	2272	2.19**	1.58–3.02	1.59*	1.14–2.23	541	2.52*	1.27–5.02	2.04*	1.01–4.12
0–4 years	849	5.35**	3.81–7.50	2.63**	1.79–3.87	1064	5.70**	2.96–10.98	3.52**	1.77–6.99
Wealth										
Highest wealth	1079	1.00	-	1.00	-	223	1.00	-	1.00	-
Second highest	1724	2.16**	1.50–3.09	1.76*	1.21–2.55	582	1.76*	1.07–2.87	1.42	0.85–2.36
Middle	633	3.08**	2.04–4.63	2.22**	1.45–3.41	418	1.69*	1.01–2.82	1.17	0.68–1.99
Second lowest	392	4.75**	3.11–7.24	3.14**	2.00–4.93	215	3.96**	2.33–6.72	2.44*	1.40–4.27
Lowest	557	6.89**	4.71–10.08	4.06**	2.65–6.22	342	3.60**	2.18–5.95	2.09*	1.22–3.56

^a^Age control: the regression includes age as control variable

^b^Full control: the model also includes residence, region, education and wealth as control variables

*p\0.05, **p\0.001

Among young women (Table 4) health was rated worse more often by rural dwellers (OR = 1.28) this did not very much change after full control. Bad SRH was more prevalent for older women in the Middle (OR = 1.98), Black Sea (OR = 1.95) and East (OR = 2.76) regions. Unlike in previous tables, this regional difference remained after full control. For education, the young group showed increased odds for middle (OR = 2.19) and lowest (OR = 5.35) groups. A similar picture was presented for the older group (OR = 2.52 and 5.70 respectively). The gradient was preserved after full control although the odds ratios were diminished. We also observed increasing odds in lower wealth groups in both age groups. After full control, the picture remained with high odds ratios (OR = 4.06 at lowest wealth group) for the younger women. For older women, an increased prevalence of bad SRH could be shown for the two lowest wealth groups (2.44 and 2.09 times).

## Discussion

### Summary of the findings

The results showed that in Turkey, substantial regional and urban/rural health differences do exist, but they can mostly be explained by differences in socioeconomic characteristics of residents. The educational level of residents and the wealth of their households are the main drivers of geographical inequalities in SRH and disability among adults in Turkey. Educational differences in health were relatively high at older ages, while the relationship of health to household wealth was stronger in younger age groups.

### Strengths and limitations

The WHS country dataset for Turkey was large enough to enable an accurate comparison between regions with regards to SRH and disability, and it was detailed enough to assess the role of socioeconomic variables. No previous study has focused specifically on regional and urban/rural inequalities in adult health within Turkey, and the contribution of individual-level socioeconomic factors.

In this study, the widely used question on SRH has been used for measuring the health status of respondents. This instrument is applicable within different cultural and socioeconomic settings. It is a strong predictor of health care utilization, morbidity and mortality outcomes [42]. However, self-reports of health outcomes are sensitive to the respondents’ perception [43] and there is evidence that disadvantaged groups may more often fail to perceive or report the presence of health problems as compared to advantaged groups [39]. If this happened in Turkey, this would have resulted in an underestimation of the true size of inequalities in SRH according to socioeconomic and geographic indicators.

As a complement to educational level, we measured the respondents’ socioeconomic status also in terms of household wealth, but not in terms of income or poverty. Household wealth was evaluated using an asset-based measurement of cumulative prosperity. This indicator has been reported to be useful for mapping health inequalities [44]. However, it is not without problems. In the Turkish setting, the increased possibility of co-residing at older ages may have biased the measurement of wealth if some of the assets counted in co-residence are not possessed by the older people. Older people moving to multi-generation households may thus be falsely measured to belong to a higher wealth category. This misclassification may have resulted in underestimation of the association between health and wealth among the older group.

This paper aimed to contribute to the understanding of regional inequalities in Turkey by assessing how much they could be explained by socioeconomic factors. We consciously did not include behaviour related variables such as smoking, physical activity and overweight, some of which we assessed in two other papers [23, 29]. The common understanding in social epidemiology is that these behavioural factors are “proximate” factors that “mediate” the association between SES and health. Thus, they are not confounders but mediators. There is no need to control for such factors in a study that focusses on the role of SES. In further analysis, we might assess whether the effect of SES is mediated by smoking and other behavioural factors, but that is beyond the scope of the current study.

The data are cross-sectional and thus can only examine associations between socioeconomic determinants and health. The results document that, at one point in time, health inequalities exist in relationship to place of residence, education and wealth. The data cannot be used to assess the direction of the causal relationships that may exist between these variables and health. The “social causation” thesis states that socioeconomic factors affect health through a number of intermediate causal pathways, while the”health selection” thesis points to processes of ‘direct’ or ‘indirect’ selection through people with poor health or low health potential are more likely to move down the social hierarchy [45, 46]. Social causation has received more empirical support than the health selection thesis.

### Why are geographical inequalities in adult health mostly small or non-existent?

If regional difference in health do exist (after adjustment for socioeconomic factors), we would have expected less favorable health outcomes for those living in underdeveloped regions than in more developed ones. But this is not consistently the case. This suggests that some dynamics of the more developed regions had affected the health of their dwellers in a negative way. One such process may relate to flows of internal migration within Turkey. 75 % of the Turkish population is now living in cities, which implies a reversal in the situation of five decades ago. Massive rural–urban migration flows may have transferred the disadvantages of the rural to the city peripheries (such as lower educational levels, the dominance of patriarchal ideology and a higher prevalence of consanguineous marriages).

Another process calls for a critical approach to the concept “development”. It is generally recognized that the more developed regions of Turkey offer more opportunities for better living and working conditions. However, those living at the outskirts of cities and working in the informal sector may face important disadvantages, including smaller social networks in the neighborhood, decreased family ties, longer time at work and transportation, and less time for leisure time activities. Such stressful circumstances make people feel worried, anxious and unable to cope [47]. In Turkish cities, high in-migration rates result in the emergence of new communities that have to struggle with the social exclusion resulting from discrimination, stigmatization, hostility and unemployment [48, 49].

Previous studies documented large regional inequalities in maternal and child health or communicable diseases [30–32]. Why did we not observe similar inequalities with regards to general adult health? One explanation may be that maternal and child health is very much dependent on sanitary conditions and access to health services, and that in these terms there are large regional and urban/rural differences in Turkey. In contrast, the key determinants of general health and disability may go beyond health services and sanitary conditions, and include a broader set of advantages and disadvantages related to stress, social exclusion and life style. Thus, it may be that while the higher levels of development improved maternal and child health in cities and Western regions thanks to improved sanitation and health services, adults’ general health was affected by a greater variety of changes in how Turkish people worked, lived and strived.

The special risks that may have arisen for the older women in the East Region may be the result of earlier life experience related to gender discrimination. In this region, the possibilities for schooling greatly differ between girls and boys and the literacy rate shows a major gender gap [50]. The gender gap in educational opportunities is exacerbated by gaps in economic and social opportunities. The dominant patriarchal ideology has brought to women, especially those with low education, higher fertility, hard physical labor, poor and delayed access to health services, and increased violence at home. Our results suggest that a life time of inequality and unease results in a higher burden of disease and disability to women at older ages.

### The changing educational and wealth-related health inequalities with increasing age

Why is education the main driver of the inequalities in health for older ages? Education mirrors individual resources and has a major role in fostering people’s own capabilities. It has effects on skills, job opportunities and earnings, and it functions as a route of transmitting resources between generations [51]. Moreover, for developing countries such as Turkey up to fifty years ago, only children from privileged families had the access to education, so that among the older generations a higher educational level is strongly correlated with privileged family background [52]. As a prominent marker of the resources early in life, a higher educational level is a key predictor of living old age in good health.

We found that inequalities according to household wealth were relatively small at older ages, yet a difference persisted between the two lowest wealth groups compared to the rest. This pattern may relate to the social security system, which distinguishes middle and higher income groups from the lowest groups. In 2006, a study by Turkish Statistical Institute found that 20 % of the Turkish people did not have any social security coverage and most of these people belonged to the poorest 30 % of the general Turkish population [53]. Having social security, a right obtained from having a previous job in the formal labor market implies a minimum wage, entitlement to health care, and a basic old-age pension. Those who are not covered and have to live on very low pension wages, are more likely to face problems such as inadequate housing and heating, insufficient nutrition, and poor access to health care. These problems add to a life of long working hours in the informal labor market with health hazards and (in case of women) exhausting child bearing and home-caring duties.

Entitlement to some source of income is essential for independence and social status, especially for the poorest elderly who have to live with other members of the family. Being a burden to other family members may cause psychological problems and may make the elderly person tend to consume less of the available family resources e.g. in case of need for health care. Contrary to elderly with high or middle income, the poor and uncovered struggle with poor access to health services [54]. Out of pocket payments in health care are higher and more frequent to the uninsured [55]. Co-payments for admission (even at primary care) drugs, transportation by private means (public transportation is not designed for disabled and unhealthy older people) place a financial burden to those who need to attend health services regularly [54].

### Conclusions

In Turkey, substantial regional differences in adult health do exist, but they can mostly be explained by differences in socioeconomic characteristics of residents and their households. These results call for a critical approach to what “development” adds and takes away to the health of residents in cities and more developed regions. The poorer health of older women in the East that persist even after control for wealth and education can be explained with their higher fertility rates and life-long exposure to gender discrimination under a patriarchal ideology. Yet, geographic differences in health are limited as compared to the large inequalities in relationship to educational level and household wealth. Not regional inequalities, but these more fundamental socioeconomic inequalities, should be of key public health concern also in Turkey.
